# Supplementary material for: Resilience across time: Longitudinal measurement invariance of the Brief Resilience Scale in French university students
Source: PLoS One. 2026 Mar 31;21(3):e0345585. doi: 10.1371/journal.pone.0345585 (PMC13037996; doi:10.1371/journal.pone.0345585)
Supplement: Attachment — Submitted filename: Response to Reviewers.docx [file pone.0345585.s002.docx]

| **Comments to the Author** |  |
| --- | --- |
| 1. Is the manuscript technically sound, and do the data support the conclusions?  The manuscript must describe a technically sound piece of scientific research with data that supports the conclusions. Experiments must have been conducted rigorously, with appropriate controls, replication, and sample sizes. The conclusions must be drawn appropriately based on the data presented. |  |
| Reviewer #1: Yes |  |
| Reviewer #2: Yes |  |
| 2. Has the statistical analysis been performed appropriately and rigorously? |  |
| Reviewer #1: I Don't Know |  |
| Reviewer #2: Yes |  |
| 3. Have the authors made all data underlying the findings in their manuscript fully available?  The [PLOS Data policy](http://track.editorialmanager.com/CL0/http:%2F%2Fwww.plosone.org%2Fstatic%2Fpolicies.action%23sharing/1/010f019a71beb329-fe9c7051-b58a-48dd-979d-efc4c762a9d5-000000/wc3quOmBc_he-Cg-Gq937X4NIaGGvdPxvUqoxy_zwrk=236) requires authors to make all data underlying the findings described in their manuscript fully available without restriction, with rare exception (please refer to the Data Availability Statement in the manuscript PDF file). The data should be provided as part of the manuscript or its supporting information, or deposited to a public repository. For example, in addition to summary statistics, the data points behind means, medians and variance measures should be available. If there are restrictions on publicly sharing data—e.g. participant privacy or use of data from a third party—those must be specified. |  |
| Reviewer #1: Yes |  |
| Reviewer #2: Yes |  |
| 4. Is the manuscript presented in an intelligible fashion and written in standard English?  PLOS ONE does not copyedit accepted manuscripts, so the language in submitted articles must be clear, correct, and unambiguous. Any typographical or grammatical errors should be corrected at revision, so please note any specific errors here. |  |
| Reviewer #1: Yes |  |
| Reviewer #2: Yes |  |
| 5. Review Comments to the Author  Please use the space provided to explain your answers to the questions above. You may also include additional comments for the author, including concerns about dual publication, research ethics, or publication ethics. (Please upload your review as an attachment if it exceeds 20,000 characters) |  |
|  |  |
| Reviewer #1: Minor edits: |  |
| 1. Lines 52-53: “University students, who are often in a period of transition and identity formation.” [Add reference/source] | A reference has been added to support this statement (Arnett, 2000), which describes university-aged individuals as being in a developmental period characterized by transition and identity exploration. |
| 2. Lines 58-60: “Studies showed that individuals with higher levels of resilience were better equipped to cope with pandemic-related stressors and reported fewer mental health symptoms (7).” [Only one reference/source, yet you mention “studies”, which I guess are multiple? Consider adding more sources, and even potentially developing slightly that insight.] | We have expanded this sentence to include multiple relevant references, as suggested. The revised text now cites additional peer-reviewed studies demonstrating that individuals with higher resilience during the COVID-19 pandemic reported lower levels of anxiety, depression, and psychological distress, supporting the plural wording of “studies.” (Killgore et al., 2020; Song et al., 2021; Barzilay et al., 2020; Lenzo et al., 2024) |
| 3. Lines 108-112: “The survey included questions on students' sociodemographic characteristics, their living and learning conditions during the lockdown, the perceived health threat of COVID-19 in their immediate environment, the impact of lockdown and related measures on their living and learning conditions, as well as self-administered questionnaires assessing their health status.” [Perhaps not necessary here in the main body, but those included questions could eventually be made accessible to the reader via a link or a supplementary material file. Or perhaps you could add something like "available via corresponding author, on demand"] | We thank the reviewer for this suggestion. We have added a statement to the manuscript indicating that the full survey questionnaire is available from the corresponding author upon reasonable request. |
|  |  |
| Moderate edits: |  |
| 4. Lines 41-42: “Psychological resilience is the capacity to adapt and recover from adversity, enabling individuals to maintain their psychological well-being in the face of stress and challenges.” [Add reference/source that clearly states that. Also, it is not a wrong definition per se, but the “maintain psychological well-being” aspect is debatable… If you “recovered” from something, can it mean that you “maintained” well-being during the whole process without it being impacted at all at any point? I would advise going slightly deeper about resilience (either in the background through your readings for a more comprehensive understanding, or here in the introduction section by adding a few sentences)] | We thank the reviewer for this insightful comment. We have revised the introductory definition of psychological resilience to include appropriate references and to clarify that resilience does not imply the absence of distress or the uninterrupted maintenance of psychological well-being. The revised text now emphasizes resilience as a dynamic process of positive adaptation, involving the capacity to maintain or regain psychological functioning following adversity, while acknowledging that temporary disruptions in well-being may occur. |
| 5. Lines 60-61: “This underscores the importance of resilience in helping individuals maintain stability during times of crisis.” [Lack of explanation here. Why precisely resilience is of help to maintain stability? Which resilience factors help, or what aspect/skill/capacity helps facing such adversity?] | We have expanded this sentence to clarify why resilience supports psychological stability during crises and added references highlighting the role of adaptive coping strategies, emotional regulation, and social support in resilient functioning. (Southwick et al., 2014; Tugade & Frederickson, 2004) |
| 6. Lines 65-66: “This has led to the development of various resilience measurement tools, such as the Brief Resilience Scale (BRS) (10). It is essential to assess whether these tools maintain consistent validity over time” [Why is the BRS the only scale mentioned? How does it represent the shift toward a modern approach to resilience (as a dynamic and evolving process)? What are the other scales available, and what are their differences? Have you considered exploring some of the advantages and disadvantages between scales in order to justify the choice of the BRS? Are this choice and these details already addressed in another publication related to the PIMS2-SPE study? If so, it would be appropriate to reference it here. Here is a link leading to literature about several measurement tools] <https://scholar.google.com/scholar?hl=fr&as_sdt=0%2C5&q=measuring+and+assessing+resilience&btnG=> | We have revised the Introduction to first situate the measurement of resilience within the broader landscape of available self-report instruments, before introducing and justifying the use of the Brief Resilience Scale (BRS). Specifically, we now briefly describe commonly used resilience scales reflecting different theoretical perspectives (e.g., the Connor–Davidson Resilience Scale and the Resilience Scale), and highlight their conceptual differences with respect to trait-based versus process-oriented approaches (Connor & Davidson, 2003; Wagnild & Young, 1993; Smith et al., 2008). We then clarify the rationale for selecting the BRS, emphasizing its focus on the ability to bounce back from stress, its alignment with contemporary dynamic conceptualizations of resilience, its suitability for longitudinal designs, and the availability of a validated French version used in student populations (Smith et al., 2008; Jacobs & Horsch, 2019, Todorovic et al., 2025). |
| 7. Lines 94-95: “The measurement times used for this study were T0 and T1.” [To me, more details on why those precise measurement times were chosen (T0 and T1) would seem important to display here.] | We agree that this required clarification. We have revised the Methods section to explicitly state that only T0 and T1 were included in the present analyses because participant attrition became substantial from T2 onward, resulting in insufficient longitudinal data to reliably conduct measurement invariance and response shift analyses. |
| 8. Lines 375-376: “The relatively short time frame between measurements also suggests that observed changes in resilience might reflect short-term adaptations, rather than long-term shifts in resilience.” [Good point here. As mentioned above, it would be interesting to add explanation on why you chose such a short time frame between the two measurements.] | We have clarified the rationale for selecting T0 and T1 in the Methods section, indicating that later follow-up waves were not included due to substantial attrition, which limited the feasibility of longitudinal invariance and response shift analyses. In addition, we have revised the Discussion to explicitly link this short measurement interval to the interpretation of the results, emphasizing that observed changes in resilience are more likely to reflect short-term adaptive responses rather than long-term shifts. |
|  |  |
| Major edits: |  |
| 9. Lines 75-76: “no previous study has assessed the longitudinal measurement invariance of resilience scales, particularly in relation to the potential effects of response shift (12,13).” [This to me seems a little hasty. More precision is needed here. Perhaps effects of response shift have not been explored previously, yet is it true for the invariance of resilience scales? Perhaps invariance in terms of effects of response shift has not been studied, but then a distinction needs to be worded here. A quick search with the right keywords made me doubt, or at least question, such lack of previous body of work you put forward here. Here is the link to literature exploring measurement invariance of resilience scales] <https://scholar.google.com/scholar?oi=gsb95&q=invariance%20resilience%20scale&lookup=0&hl=fr> | We have revised the relevant sentence to more precisely distinguish between existing work on cross-sectional measurement invariance of resilience scales and the specific gap targeted by our study. The revised text now acknowledges that some resilience measures (e.g., CD-RISC-10 and BRS) have demonstrated cross-sectional measurement invariance across groups, citing appropriate references (Kavcic et al., 2021; Tu et al., 2023; Saha et al., 2025; Todorovic et al., 2025). We then clarify that, to our knowledge, few studies have examined longitudinal measurement invariance of resilience scales, and none have explicitly addressed the potential effects of response shift on their longitudinal validity during prolonged stressors such as the COVID-19 pandemic. |
| 10. Lines 80-81: “We hypothesized that the BRS, when used across multiple time points, would maintain its psychometric properties, enabling valid comparisons of resilience over time.” [Why such a hypothesis? On what elements and/or theoretical and empirical insights do you base your hypothesis on?] | We have revised the manuscript to explicitly justify this hypothesis. The Introduction now clarifies that our expectation of longitudinal stability was based on prior psychometric evidence supporting the BRS’s unidimensional structure, internal consistency, and stability across populations and contexts, as well as its conceptualization of resilience as a general recovery capacity. Relevant references have been added. (Smith et al., 2008; Jacobs & Horsch, 2019; Todorovic et al., 2025) |
| 11. Recap for the whole introduction section: [I have not checked the author guidelines; therefore, I don't know if you would have room and could be allowed for a lengthier introduction... Yet if so, I would globally consider adding more depth to this whole introduction section... - What are the different tools/scales for measuring resilience? - Or at least, what are some of the more robust and well-established ones? - What are their advantages/disadvantages, or what are some of their differences? - What are the ones/the one that align the best with modern approaches considering resilience as a dynamic process? - Why choosing the BRS?] | We thank the reviewer for this thoughtful and integrative comment. In response, we have strengthened the Introduction by briefly situating the Brief Resilience Scale (BRS) within the broader landscape of well-established resilience measures (e.g., CD-RISC, RS), highlighting their key conceptual differences, and clarifying how these instruments align with trait-based versus process-oriented approaches to resilience. We have also expanded the rationale for selecting the BRS, emphasizing its conceptual consistency with contemporary dynamic definitions of resilience, its psychometric robustness, and its suitability for longitudinal assessment in student populations during the COVID-19 pandemic. While we aimed to keep the Introduction concise and focused on the specific objectives of the present study, we believe these additions provide sufficient depth to justify the measurement choice without extending beyond the scope of the article. |
| 12. Lines 323-324: “This aligns with earlier studies on resilience measurement, which suggest that while resilience is a dynamic process that can evolve over time, it is also stable enough to be measured consistently across time points (10).” [This needs to be more developed and discussed. Earlier studies on resilience measurement have not been mentioned (nor reflected upon) enough throughout the paper (introduction, and here in the discussion). I would advise to at least give some insights here on the results and implications from those earlier studies in order then to compare them to your own results and in order to offer a more comprehensive and complete discussion. Also there needs to be more clarity on the fact resilience is said to be a dynamic process while being stable enough to be measured across time points. To me your assertion here seems too hasty and needs to be developed and explored.] | We thank the reviewer for this important comment. We have revised this section of the Discussion to more explicitly reflect on prior psychometric research on resilience measurement. The revised text now clarifies how earlier studies have shown that resilience scales can exhibit stable measurement properties (e.g., factor structure, reliability, and invariance) despite resilience being conceptualized as a dynamic process. We also expanded the discussion to explain how measurement stability does not contradict conceptual dynamism, and we explicitly position our findings as extending this literature by demonstrating longitudinal measurement invariance and absence of response shift in a crisis context. |
| 13. Recap for the whole discussion section: [This discussion section could point to some missing elements/aspects in your introduction (elements that perhaps you'll add in the intro so that you'll be able to link them to your discussion here). More globally, it could be interesting to explore some of the following questioning: - Do all resilience scales encapsulate the dynamic nature of resilience? - How does the BRS position itself in this regard? - What dimensions/factors of the BRS and of other well-known scales (in those earlier studies maybe) account for the dynamism of the resilience construct? - At the same time, how come those dimensions/factors/items account for some sort of stability in the results across time points?] | We thank the reviewer for this integrative comment. In response, we have expanded the Discussion to more explicitly address how resilience scales differ in their conceptualization of resilience and how this relates to the dynamic versus stable aspects of the construct. The revised text now clarifies that not all resilience measures equally capture resilience as a dynamic process, and it situates the Brief Resilience Scale as a recovery-focused instrument designed to reflect adaptive change while maintaining stable item meaning over time. We further explain how this measurement approach allows resilience levels to vary across time points without introducing response shift, thereby strengthening the coherence between our Introduction, Discussion, and empirical findings. |
| 14. GENERAL AND/OR ADDITIONAL COMMENT: [I sure understand you are not writing a theoretical paper about resilience, neither that you aim at discussing conceptual boundaries of such a construct and the tools developed to measure it, so no worries here. You do not need to rethink the whole approach behind your study. My point is that, to me, a more comprehensive reflection and a deeper discussion need to be displayed in your paper about these aspects (results from earlier studies; comparison with your results; dynamism of resilience while scales lead to stable results; resilience dimensions targeted within those measuring tools; and how all of these insights are to be interpreted altogether...)] | We thank the reviewer for this thoughtful and integrative comment. In response, we have strengthened both the Introduction and the Discussion to provide a more explicit and coherent reflection on the conceptual and psychometric aspects of resilience measurement, without shifting the focus of the paper toward a purely theoretical discussion.  Specifically, we expanded the Discussion to (i) situate our findings more clearly in relation to earlier psychometric studies of resilience scales, (ii) compare these prior results with our own longitudinal and response-shift–informed findings, (iii) explicitly address how resilience can be conceptualized as a dynamic process while still yielding stable measurement properties, and (iv) clarify how different resilience instruments target distinct dimensions of resilience (e.g., trait-based resources versus recovery capacity), with particular emphasis on the positioning of the BRS.  These additions were intended to offer a more integrated interpretation of our results, linking theory, measurement, and empirical findings, while remaining consistent with the applied and psychometric scope of the study. We hope this revised version more clearly reflects the depth of reflection requested. |
|  |  |
| Reviewer #2: The ethical standards of the study were respected (according to the principles of the Declaration of Helsinki). The study framework, the composition of the study population sample, and the data collection and processing were carried out rigorously and clearly explained within the article. The bibliographic references are robust. The results were thoroughly analyzed, and the data interpretation was performed accurately and cautiously. In conclusion, this article demonstrates sound scientific quality and is suitable for publication as is. | We sincerely thank the reviewer for their careful evaluation of our manuscript and for their positive and encouraging feedback. We appreciate the reviewer’s assessment of the ethical rigor, methodological clarity, robustness of the references, and the cautious interpretation of the results. We are grateful for their conclusion that the manuscript demonstrates sound scientific quality and is suitable for publication. |
